# Non syndromic synchronous multiple odontogenic keratocysts in a western Indian population: A series of four cases 

**DOI:** 10.4317/jced.54616

**Published:** 2018-08-01

**Authors:** Krishna-Sireesha Sundaragiri, Shikha Saxena, Bharat Sankhla, Akshay Bhargava

**Affiliations:** 1 MDS, Assistant Professor, Department of Oral Pathology, RUHS College of Dental Sciences, Jaipur, Rajasthan, India

## Abstract

Odontogenic keratocysts (OKCs) are developmental odontogenic cysts affecting the maxillofacial region and their association with a syndrome especially Naevoid basal cell carcinoma syndrome (NBCCS) is a common occurrence in comparison to non syndromic multiple OKCs. In a first, we present a series of four non syndromic cases with multiple OKCs in western Indian population. The presence of multiple OKC in our present case series may be because of the multifocal nature of the lesion rather than its association with any syndrome. Thus, a comprehensive evaluation of any patients reporting with multiple cysts/OKCs always has to be undertaken and the dental practitioner may be the play a key role in early detection and subsequent follow-up.

** Key words:**Abnormalities, multiple; basal cell nevus syndrome, India, odontogenic cysts.

## Introduction

Odontogenic Keratocysts (OKC) has been in past synonymously known as primordial cysts, keratocystic odontogenic tumour (KCOT) (1) is an odontogenic cyst characterized by a thin, regular lining of parakeratinized stratified squamous epithelium with palisading hyperchromatic basal cells. Speight P *et al.*, in the new 2017 WHO Classification of Head and Neck Tumours, re-instated the term ‘Odontogenic Keratocyst’. The authors have also stated that ‘further research is needed, but at the present time there appears to be insufficient evidence to support a neoplastic origin of OKC. It was therefore felt that OKC remains the most appropriate name for this lesion’ (2).

OKCs account for 10-20% of odontogenic cysts and are the third most common cyst of the jaws (2) but the simultaneous occurrence of multiple cysts in both the maxilla and mandible of a patient is rare. About 10% of patients have multiple OKCs (either metachronous or synchronous), and half of these patients have Naevoid basal cell carcinoma syndrome (NBCCS) (2). Other syndromes with which multiple OKCs have been described are orofacial digital syndrome (OFDS) (3), Ehler-Danlos syndrome (EDS) (4,5), Noonan syndrome (6,7) , Simpson-Golabi-Behmel syndrome (8). There is no specific laboratory test to diagnose NBCCS and the diagnosis is made clinically using the specific criteria suggested by the first international colloquium on basal cell nevus syndrome (BCNS) ( Table 1). It was decided that a suspected diagnosis of BCNS/NBCCS could be reasonably considered based on the findings of less stringent criteria of: (i) one major criterion and molecular confirmation; (ii) two major criteria; or (iii) one major and two minor criteria (9).

**Table 1:**
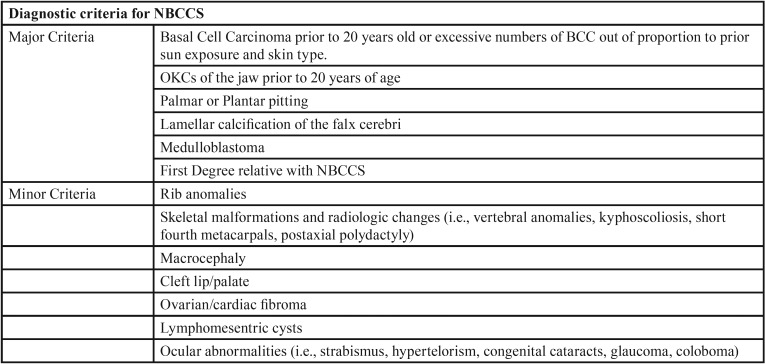
Diagnostic criteria for NBCCS based on the consensus statement from the First International Colloquium on NBCCCS (9).

Gupta SR *et al.*, noted that only 23 cases of NBCCS have been reported in Indian patients, it is evident that the frequency of clinical and radiological features in NBCCS in Indian patients differs from other ethnic groups. Either NBCCS is rare in the Indian population or may be under-reported owing to lack of awareness about the clinical and radiological manifestations of NBCCS. The absence of family history and other features of the syndrome can be due to variation in penetration and expression of different mutations within the Patched (PTCH) gene or the effects of modifier genes and environmental factors in different ethnic groups (10).

No specific etiology for the formation of multiple cysts has been defined as such. Multiple OKCs may occur without the syndrome probably as a result of the multifocal nature of lesion and not always because of genetic defect. Boyne *et al.*, suggested that dramatic mutations in PTCH gene could result in activation of the various resting state OKC epithelial cells of the primary lesion or the involved bone – ‘multifocal nature’. The PTCH gene when present exerts its protective influence in the non syndromic sporadic cases of OKCs as well (11).

Multiple radiolucencies in the jaws can also be formed by various other pathologies such as and should be differentiated from multiple cystic lesions (12). OKCs radiographically present as a well-defined multilocular or unilocular radiolucent lesion. Multilocular radiolucency more frequently seen for mandibular lesions similar to ameloblastoma (13). A radiolucent lesion associated with a tooth - unerupted or impacted is not distinguishable from a dentigerous cyst is also common presentation for OKC. Concomitant occurrence of multiple OKCs with other dental anomalies such as transposition, short dilacerated roots may be coincidental (14).

The non syndromic or syndromic OKCs are lined by characteristic keratinizing stratified epithelium. Treatment usually involves surgical enucleation and chemical cauterization with a mild, not deeply penetrating, cauterizing agent as Carnoy’s solution and marsupialization or surgical resection is case of larger cysts. There is no evidence of any difference in behaviour between syndromic and sporadic OKCs and the management is the same (2). Multiple OKCs unassociated with any syndrome have been reported rarely.

## Case Report

So as to add to the growing number of such cases in the literature here we have reported a series of four cases of non syndromic synchronous multiple OKCs reported to department of Oral Surgery, RUHS College of Dental Sciences, Jaipur. Written patients’ consent was obtained for history, biopsy and subsequent surgery. Any relevant past history or family history of cysts/tumours was carefully noted for each patient. The presence of multiple cyst-like lesions on radiological examination at the time of first presentation of each case and their subsequent histopathological examination defined these cases as synchronous multiple lesions. After extra-oral and intra-oral clinical examinations, orthopantomogram (OPG), Computed tomography (CT) and radiological evaluation of skull bones, chest, hands, feet, long bones, pelvis, and spine were carried out for all patients. The patients were referred to dermatology (for cutaneous abnormalities), neurology (cranial abnormalities), ophthalmology (for ocular abnormalities), cardiology, surgery and ear, nose, and throat departments for a multisystem evaluation. The processing and histopathological examination of the biopsy specimen was done in the Department of Oral Pathology.

-Case 1

**Figure 1:**
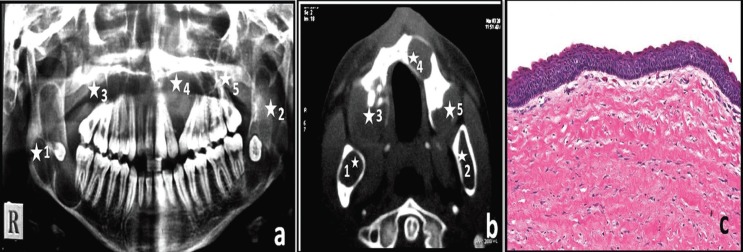
Multiple cyst-like lesions of jaws seen in OPG(a) and CT(b) of Patient 1 (white asterisk indicate 5 lesions in a single patient). Photomicrograph showing H& E stained typical corrugated parakeratinized stratified epithelium diagnostic of OKC (c) at 10x magnification.

A 15 year old male patient with a history of swelling in right and left back region of mouth since last 18 months visited the OPD. Intra-oral examination showed approximately 3 x 2.5 cm swelling in the right lower side and 3 x 3 cm swelling in the left lower side. Patient was not aware of any maxillary lesions as the radiographical examinations revealed multiple radiolucent (05) lesions: i) multilocular radiolucent lesion distal to roots of 46 up to ramus involving impacted 48, ii) multilocular radiolucent lesion distal to roots of 37 upto ramus involving impacted 38, iii) unilocular radiolucent lesion distal to roots of 17, iv) unilocular radiolucent between displaced roots of 22, 23, v) unilocular radiolucent lesion distal to roots of 27 (Fig. 1a,b) Provisional diagnosis of OKC for the three maxillary lesions, OKC or dentigerous cysts was made in case the two mandibular radiolucencies was associated with an impacted tooth. The incisional biopsy of the two larger mandibular lesions was done and sent in separate bottles for histopathological diagnosis. Characteristic features of uniform thick parakeratinised epithelium, basal cell palisading resembling picket fence appearance indicated an OKC diagnosis for both the lesions. A thorough examination for any sign of NBCCS, considering the age of the patient where the multiple cysts could be the first sign of the syndrome was advised. Under general anesthesia, based on the size of the cysts, enucleation of maxillary cysts was done followed by chemical cauterization. Considering the extent of the two mandibular lesions and age of the patient marsupialization was done for mandibular cysts and subsequent removal of the lesion along with the impacted third molars was done and sent for histopathological examination. The two mandibular lesions and two maxillary lesions where diagnosed as OKC (Fig. 1c)while the left posterior maxillary lesion was diagnosed as orthokeratinized odontogenic cyst. The patient was followed up for 2 years without any signs of recurrence or any other sign of NBCCS.

-Case 2

**Figure 2:**
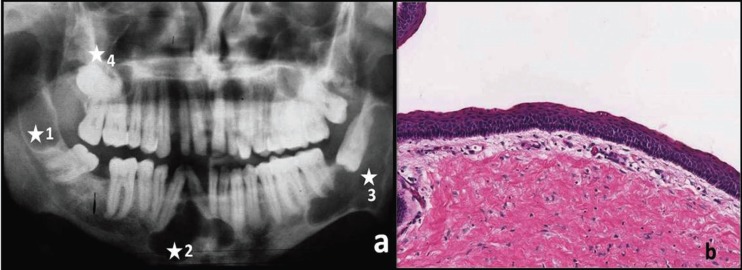
Multiple cyst-like lesions of jaws seen in OPG (a) of Patient 2 (white asterisk indicate 4 lesions in a single patient). Photomicrograph showing H& E stained typical corrugated parakeratinized stratified epithelium and prominent basal cell layer is diagnostic of OKC (b) at 10x magnification.

A 21 year old female patient reported with complaint of swelling in the lower anterior region of mouth since last 6 months. Patient also complained of crowding of lower right teeth. Intra oral examination revealed 2 x 3cm swelling in the lower left region with missing 38. Radiographic examination showed multiple radiolucent (4) lesions. i) unilocular radiolucent lesion distal to roots of 48 upto ramus with missing 47, ii) multilocular radiolucent lesion extending from 43 to 32 also causing the root divergence of 43 and 42, iii) multilocular radiolucent lesion with scalloped margins along the roots of 36, 37 to ramus involving impacted 38, iv) unilocular radiolucency along roots of 16, 17 with impacted 18 (Fig. 2a). A provisional diagnosis of ameloblastoma/OKC for right posterior and anterior mandibular lesions, dentigerous cyst/ enlarged dental follicle was made in case of right maxillary posterior and dentigerous cyst/ unicystic ameloblastoma for left mandibular posterior radiolucency that were associated with an impacted tooth. Incisional biopsy was carried out for the left and right posterior lesions and right anterior region while the right maxillary posterior cystic lesion was removed in toto along with impacted 18. The biopsies were sent in separate labeled bottles for histopathology examination and all were diagnosed as OKCs (Fig. 2b). A thorough examination for any sign of NBCCS was carried out. Surgically under general anesthesia, based on the size of the cysts, enucleation of right mandibular anterior and posterior cysts was done followed by chemical cauterization. Considering the extent of the left posterior mandibular lesion and age and sex of the patient marsupialization was done with subsequent removal of the lesion. The histopathological examination of all lesions was diagnosed as typical OKCs. Post surgery the patient was followed up for two year without any signs of recurrence or any syndrome.

-Case 3

**Figure 3:**
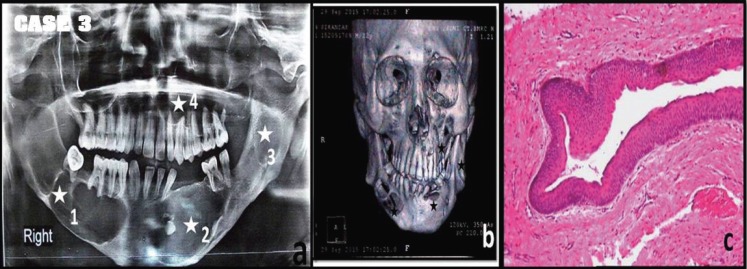
Multiple cyst-like lesions of jaws seen in OPG(a) and 3D-CT(b) of Patient 3 (white asterisk indicate 4 lesions in a single patient). Photomicrograph showing H& E stained typical hyper parakeratinized stratified epithelium, typical infoldings and keratin flakes in the lumen diagnostic of OKC (c) at 10x magnification.

A 25 year old male patient visited the OPD with a complaint of fluctuant swelling in the lower right of mouth since last 18 months region. Intra oral examination showed approximately 4 x 3 cm fluctuant swelling on the right posterior mandibular region with egg shell crackling and an approximately 2 x 3 cm swelling in the left anterior mandibular region. The radiographical examination revealed multiple (04) radiolucent lesions: i) multilocular radiolucency in the mandibular angle with scalloped margins in 44, 45, 46 & 48 region, root resorption of 44, 45, 46 & 48 seen and missing 47. ii) unilocular radiolucency extending from 42 to 38 with impacted 33 and missing 41, 35, 36 and transposed tooth 34 along with root resorption. iii) unilocular radiolucency in left mandibular ramus, iv) multilocular radiolucency along roots of 21 to 27 (Fig. 3a). The CT showed bone perforations below roots of 43, 44, 45, 46 on the right side and 33 on the left side (Fig. 3b). A provisional diagnosis of OKC/ dentigerous cyst/ ameloblastoma was given. Incisional biopsy was performed for the mandibular lesions and left posterior maxillary lesion. The histopathological examination revealed the diagnosis of typical OKCs. The surgical treatment of enucleation followed by chemical cauterization for all the lesions were carried out under general anesthesia and the specimens sent in separate labeled bottles. The final histopathological diagnosis of all the lesions was OKC (Fig. 3c). The patient was informed about developing the possibility of any other signs and symptoms of NBCCS. The patient was followed up for 18 months without any signs of recurrence or NBCCS.

-Case 4

**Figure 4:**
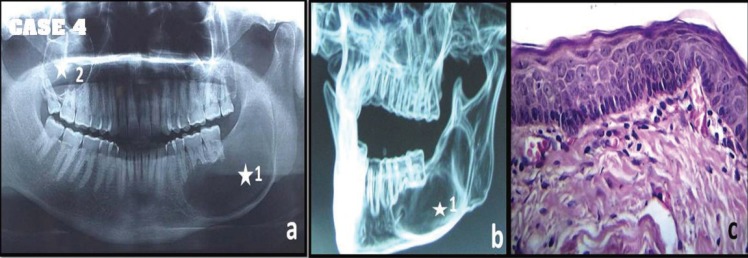
Multiple cyst-like lesions of jaws seen in OPG(a) and contrast enhanced-CT(b) of Patient 4 (white asterisk indicate 2 lesions in a single patient). Photomicrograph showing H& E stained typical hyper parakeratinized stratified epithelium with prominent basal cell layer is diagnostic of OKC (c) at 40x magnification.

A 35 year old male patient reported with the complaint of recurrent swelling in the left lower side of face since last 12 months with history of extraction of lower left third molar. The intra-oral examination revealed 4 x 3 cm swelling in left mandibular posterior region. The radiographic examination showed multiple (2) radiolucent lesions: i) multilocular radiolucent lesion next to teeth 35, 36, 37 extending upto ramus region and root resorption of 35, 36, 37 noted ii) multilocular radiolucent lesion along roots of 16, 17 & 18 with root divergence of tooth 18 (Fig. 4a,b). After a thorough clinical examination, incisional biopsy of the two lesions was done and the specimen sent in separate labeled bottles. The histopathology examination revealed the two cystic lesions as OKC. The patient underwent surgical enucleation of both the lesion along with removal of 18 tooth and specimen sent for histopathological examination. The examination revealed OKCs in the excision biopsy specimen (Fig. 4c). The patient was very circumspect and apprehensive about the possibility of syndrome and was free of recurrence and any syndrome after 2 years of follow-up.

## Discussion

Multiple OKCs might be the first and the only manifestation of NBCCS without any other features associated with syndrome. However, other symptoms can occur in later decades of life. The appearance of two or more recurrent OKCs or the appearance of an OKC in a young patient should lead dental practitioners to suspect that the patient has NBCCS (15). Patients with multiple OKCs with or without NBCCS, are generally younger than those with single OKCs (16). As in the context of Case 1 who is the youngest in our series, a long term has been planned for him whereas Case 2 and Case 3 are in the third decade of life which is the one peak age of incidence of OKCs (2). All our cases were lined by characteristic keratinizing stratified epithelium.

The current 2017 WHO classification categorically states about the much debated discussion of the distinction between non-neoplastic and neoplastic cystic lesions of OKC/ KCOT and the calcifying cystic odontogenic tumour (CCOT)/ calcifying odontogenic cyst (COC) and persisted in the time-honored terms of OKC and COC as the editors felt that most cases of KCOT and CCOT behave clinically as non-neoplastic lesions and are treated as cysts (2). This case series too supports this view that sporadic multiple OKCs tend to behave more like developmental cysts than benign tumours.

The multifocal nature of OKC may the source of occurrence of multiple cysts as discussed by Boyne in their study of resected mandibular specimens with OKCs (11). This paper highlights that any patient reporting with the multiple OKCs should be evaluated thoroughly for the possibility of NBCCS as multiple OKCs can occur a decade before other symptoms associated with NBBCS and clinical manifestations of NBCCS may remain hidden in the earlier years of life and there is continued development of new and recurring cysts until about age 30 (17). Thus a general dental practitioner may well be the first to detect as well as maintain a strict follow-up of the syndrome.
